# Comparison and Analysis between the NAV6 Embolic Protection Filter and SpiderFX EPD Filter in Superficial Femoral Artery Lesions

**DOI:** 10.1155/2021/9047596

**Published:** 2021-06-01

**Authors:** Prakash Krishnan, Arthur Tarricone, Allen Gee, Serdar Farhan, Haroon Kamran, Annapoorna Kini, Samin Sharma

**Affiliations:** The Zena and Michael A. Weiner Cardiovascular Institute and the Marie-Josée and Henry R. Kravis Cardiovascular Health Center, Department of Medicine/Cardiology, Icahn School of Medicine at Mount Sinai, New York, NY, USA

## Abstract

**Objective:**

To compare the safety and efficacy between the SpiderFX EPD and Emboshield NAV6 filter in the collection of embolic debris created from lower limb atherectomy procedures in patients with PAD.

**Materials and Methods:**

Between January 2014 and October 2015, 507 patients with symptomatic peripheral artery disease were treated with directional atherectomy (SilverHawk), rotational atherectomy (JetStream), or laser atherectomy (Turbo Elite) based on operator discretion. Emboshield NAV6 (*n* = 161) and SpiderFX (*n* = 346) embolic protection devices were used with each of the 3 atherectomy devices. The primary study endpoint was 30-day freedom from major adverse events (MAEs). An MAE was defined as death, MI, TVR, thrombosis, dissection, distal embolization, perforation at the level of the filter, and unplanned amputation. A descriptive comparison of the MAE rates between Emboshield NAV6 and SpiderFX embolic protection devices was conducted.

**Results:**

The freedom from major adverse event (MAE) rate was 92.0% (CI: 86.7%, 95.7%) in patients who received an Emboshield NAV6 filter compared to 91.6% (CI: 88.2%, 94.3%) in patients who received the SpiderFX filter (*p*=0.434). The lower limit of 86.7% freedom from major adverse event rate in the Emboshield NAV6 group was above the performance goal of 83% (*p* < 0.0008).

**Conclusions:**

There were no significant clinical outcome differences between Emboshield NAV6 and SpiderFX EPD filters in the treatment of lower extremities. This evaluation indicates the safety and efficacy to use either filter device to treat PAD patients with lower extremity lesions.

## 1. Introduction

Peripheral artery disease (PAD), defined as the atherosclerotic disease of the lower extremity, affects over 200 million people worldwide, and its prevalence rises in aging populations [1–4]. The use of atherectomy in endovascular treatment may cause distal embolization (DE) [5]. Despite DE occurring at a relatively low incidence, clinical concern remains over its potential to adversely affect several patient outcomes including, but not limited to, distal outflow, increase in reinterventions, amputations, increase in procedure time, prolonged hospital stay, and increase diagnostic, pharmacy, and laboratory costs [6–8].

Knowing the negative outcomes associated with DE, the use of filters or embolic protection devices (EPDs) originally used in carotid and saphenous vein coronary bypass grafts has become popular in peripheral interventional procedures. The use of current Food and Drug Administration- (FDA-) approved EPDs in the lower extremities such as the SpiderFX EPD and WIRION has been shown to be effective in reducing the rates of major adverse events (MAEs) [9]. The Emboshield NAV6 Embolic Protection System (Emboshield NAV^6^) is an EPD that is approved for carotid use and has demonstrated its efficacy and safety [10, 11]. To date, Emboshield NAV^6^ has been used as an off-label EPD in lower extremity procedures, but has yet been demonstrated to be safe and efficacious [12–15]. The objective of this study was to evaluate and compare the safety and efficacy of the Emboshield NAV6 with the currently approved SpiderFX filter. A comprehensive database of PAD patients treated at a single center where both the Emboshield NAV6 and SpiderFX devices were used provided the necessary procedural and safety data to complete the study objective. The study's primary objective was to compare the safety and efficacy of the Emboshield NAV6 filter with the SpiderFX EPD filter in the collection of embolic debris created during lower limb endovascular procedures in patients with PAD.

## 2. Methods

### 2.1. Study Design and Patient Population

This study is a retrospective analysis of real-world data from patients with symptomatic lower extremity PAD who were treated with atherectomy and distal embolic protection between January 2014 and October 2015. Patients with femoropopliteal lesions above the P2 segment and all Trans-Atlantic Inter-Society Consensus (TASC) classification types were included. The study excluded patients with inflow disease that affected the iliac arteries. Previous work described using this database included clinical, demographic, and angiographic data to develop an algorithm for embolic protective device (EPD) use [16].

Patients provided informed consent at the time of the procedure. The hospital received institutional review board (IRB) approval from the Program of the Protection of Human Rights to share these data with the sponsor for this investigation without the requirement for additional informed consent. In addition, the data analysis proposed by the sponsor was approved by the Program of the Protection of Human Rights (HS# 18-00707).

### 2.2. Study Treatment

Study treatments were previously described in detail [17]. Briefly, patients with PAD, specifically with femoral popliteal lesions, were treated with either directional atherectomy (SilverHawk), rotational atherectomy (Jetstream), or laser atherectomy (Turbo Elite), based on operator discretion. Both Emboshield NAV6 and SpiderFX EPDs were used with each of the 3 atherectomy devices, per operator discretion. The EPD was placed before the atherectomy procedure and was in place for all patients. The filter was placed at the level of the popliteal artery.

### 2.3. Clinical Endpoints

A major adverse event (MAE) was defined as a composite of death, myocardial infarction (MI), thrombosis, DE, dissection (grade C or greater), perforation at the level of the filter, amputation, and target vessel revascularization (TVR). The 30-day primary endpoint of freedom from MAE was analyzed against a performance goal (PG). The PG was based on historical data from the DEFINITIVE Ca^++^ trial [9]. Procedural analyses were also reported, including successful delivery of the filter, quantity of filters with macroemboli, and freedom from device malfunction (for example, failure to deploy/advance, kinking of wire, and device component detachment), as available in the database. Subset analyses included calcified, chronic total occlusion (CTO) and in-stent restenotic lesions.

### 2.4. Statistical Analysis

Descriptive analysis was performed without hypothesis testing on variables reflecting demographic information, baseline risk factors, and procedural/angiographic characteristics, including the success of filter delivery, device malfunction, presence of macroemboli, and cases of filter overflow. A descriptive analysis was conducted comparing the Emboshield NAV6 and SpiderFX EPDs demographic information, baseline risk factors, and procedural/angiographic characteristics and 30-day MAE rate. Descriptive, nonpowered subset analyses were conducted on outcomes for all lesion types available from the dataset. Subset analyses were nonprespecified, nonpowered, and descriptive only.

Continuous variables were presented as mean ± standard deviation, while categorical variables were presented as percentages. Pearson *X*^2^ and independent *T*-tests were used as appropriate to compare variables. *p* < 0.05 was considered significant. All calculations were performed using the SPSS statistical software package (version 20.0; IBM Corporation, Somers, NY).

## 3. Results

### 3.1. Patient Disposition

**Figure 1:**
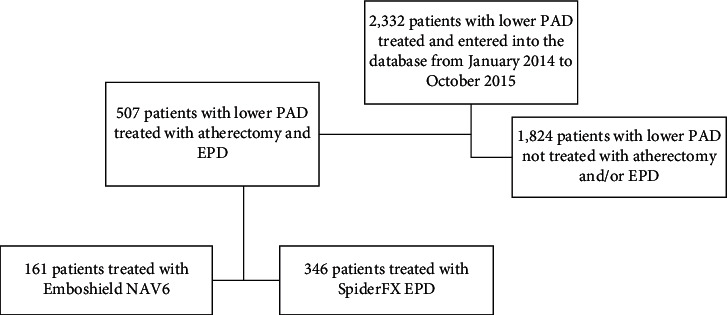
Flowchart of patients included in this study. EPD, embolic protection device; PAD, peripheral artery disease.

From January 2014 to October 2015, data from 2,332 patients treated with lower extremity PAD were collected at a single center.  Figure 1 outlines the selection of 507 patients—161 received Emboshield NAV6, and 346 received SpiderFX EPD—whose data were analyzed. Follow-up was conducted to 30-days after procedure on all 507 patients, per hospital standard of care.

### 3.2. Patient Demographics and Baseline Risk Factors

Patient demographics and baseline risk factors were similar between Emboshield NAV6 and SpiderFX groups (Table 1). The patient population in both groups was approximately 70 years old, with a nearly even distribution of males and females. Overall, this was a metabolically diseased population in which most patients in both groups had overweight or obesity (average BMI close to 28 kg/m^2^); majority had hyperlipidemia, hypertension, and coronary artery disease; approximately 60% had diabetes; and nearly half of patients smoked. Both groups also had creatinine levels slightly above normal, and there was a small percentage of chronic kidney disease.

### 3.3. Baseline Lesion Characteristics

Treatments were in the SFA, with the exception of 2 in the femoropopliteal artery for each treatment group. No significant differences were found in lesion length or reference vessel diameter (RVD) between the groups (Table 2). Lesions in both groups were complex in nature with a high average lesion length and the presence of calcification and CTOs. The frequency of preprocedure in-stent restenosis was 6.8% in Emboshield-NAV6-treated patients and 8.1% in the SpiderFX group.

### 3.4. Procedural Results

There were some procedural differences as noted in Table 3. The majority of patients were treated with directional atherectomy, and the remainder was roughly equally between rotational atherectomy (Jetstream) and laser atherectomy (Turbo Elite). Access complications were related to closure devices and not related to filter therapy or treatment and were reported in 2 patients in the SpiderFX group. Recoil was related to balloon treatment and was reported in 4 patients in the Emboshield NAV6 group. As expected, patients had high preprocedure stenosis and low postprocedure stenosis, indicating a successful percutaneous procedure.

Approximately 10% of patients receiving Emboshield NAV6 filters experienced filter overflow, defined as macroscopic debris filling the entire filter with no flow through the filter after intervention (1). This was not specific to Emboshield NAV6, as a similar rate was observed with SpiderFX EPD. Macroemboli was present in close to 60% of Emboshield NAV6 filters and close to 64% of SpiderFX filters (*p*=0.352).

All Emboshield NAV6 filters were delivered successfully. There was 1 case of perforation and 2 cases of thrombus in Emboshield-NAV6-treated patients. The single case of perforation was the only procedure-related complication documented for all Emboshield NAV6 patients, and it was caused by migration of the wire into the filter. The complication was treated with prolonged balloon inflation without sequelae. Therefore, 159/162 of the Emboshield NAV6 had no complications resulting in a 99% freedom from procedural complications rate. All filters were delivered successfully, and there were no device malfunctions.

### 3.5. Primary Endpoint

**Figure 2:**
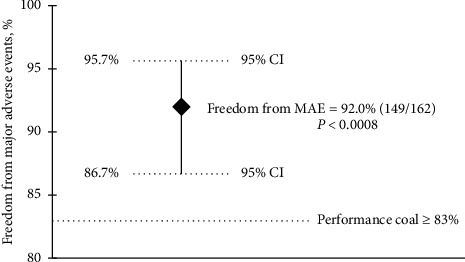
Freedom from major adverse events through 30 days in patients receiving the Emboshield NAV6 filter. CI, confidence interval; MAE, major adverse event.

The primary safety and effectiveness outcome evaluated were freedom from MAE (Figure 2), which was 92.0% (CI 86.7%, 95.7%) for Emboshield NAV6 and 91.6% (CI: 86.2%, 96.7%) for SpiderFX (*p*=0.721) (Table 4). The lower limit of 86.7% was above the predefined PG of 83% (*p* < 0.0008). Therefore, the performance goal for the primary endpoint was met.

### 3.6. Event Data

In the Emboshield NAV6 group, 13 events contributed to the MAE rate: 1 death, 2 MIs, 1 thrombosis, 8 dissections, 1 distal embolism (DE), and 1 perforation at the level of the filter or unplanned amputations. In the SpiderFX group, 29 events contributed to the MAE rate: 1 death, 10 MIs, 4 thrombosis, 10 dissections, and 3 DE (Table 4).

There was 1 death in each group; neither patient experienced any other adverse event, and details of the deaths were lacking in the database.

There were 2 cases of MI in the Emboshield NAV6 group and 10 in the SpiderFX group. In both MI cases for Emboshield NAV6 and 9/10 cases for SpiderFX, no date was available for when the MI occurred. The events were assumed to occur within 30 days and included in the MAE rate. Thrombosis was reported in 1 patient receiving an Emboshield NAV6 filter and 4 patients receiving SpiderFX filters. All events, except for 1 SpiderFX EPD case, occurred on the day of the procedure. The remaining thrombosis event in the SpiderFX EPD group was reported 1 week after procedure along with TVR. Another event of TVR occurred in a SpiderFX EPD patient and was reported approximately a month later.

Dissections occurred in 8 Emboshield NAV6 cases and 10 SpiderFX cases. Dissection is typically a complication related to angioplasty balloon expansion and is not a complication typically associated with EPD. The total number of DE across both groups was 4.

### 3.7. Subset Analysis

The lesion types available for analysis in the Emboshield NAV6 dataset were calcified lesions, CTOs, and restenosis lesions.

#### 3.7.1. Calcified Lesions

Subset analysis showed that 70 patients with calcified lesions treated with Emboshield NAV6 had a freedom from MAE rate of 88.6% compared to a 94.6% rate in 90 patients without calcified lesions (Table 5). The components of MI, thrombosis, and DE were similar across lesion types. All 8 observed dissections occurred in calcified lesions. Analysis in the 131 patients with calcified lesions treated with SpiderFX had a freedom from MAE rate of 95.4% compared to 91.7% in the 216 patients without calcified lesions (Table 5). All clinical outcomes were similar across lesion types except for dissection, where the rate was greater in the noncalcified SpiderFX cohort.

#### 3.7.2. CTO Lesions

Chronic total occlusion was defined as 100% occlusion at any point within the SFA or popliteal artery. In the Emboshield NAV6 dataset (Table 6), there were 46 patients with CTO lesions with a freedom from MAE rate of 84.8%; the 115 patients with non-CTO lesions had a freedom from MAE rate of 94.8%. Similar to the calcified lesions, a higher number of dissections occurred in the CTO group. In the SpiderFX dataset (Table 6), there were 95 patients with CTO lesions with a freedom from MAE rate of 88.4%; the 251 patients with non-CTO lesions had a freedom from MAE rate of 92.4%. Unlike the NAV6 dataset, dissections along with all other clinical outcomes were similar across both CTO groups and non-CTO groups.

## 4. Discussion

As endovascular procedures become the mainstay of treatment for symptomatic PAD, EPDs are often used to prevent the complications caused by DE [18]. In an earlier endovascular trial comparing the presence and absence of EPDs, the non-EPD group experienced twice as many DE in comparison to the EPD cohort [6]. It has also been documented that atherectomy and stent deployment induce DE more than percutaneous transluminal angioplasty alone [19]. The occurrence of DE can adversely affect distal outflow, lead to increased reinterventions and amputations, increase procedure time, prolong hospital stay, and increase diagnostic, pharmacy, and laboratory costs [6–8]. At the time of this study, only the SpiderFX was indicated for the prevention of DE in calcified lower extremity lesions in conjunction with atherectomy. However, endovascular interventionalists have begun to adopt the use of Emboshield NAV6 for many reasons including the ability of having greater manipulation of the device during procedure.

While the safety and efficacy of Spider FX has been well established and leads to indication in the use of atherectomy for calcified femoropopliteal disease, Emboshield offers several advantages. These include the use of BareWire® technology that allows the Emboshield wire and filter to move independently of each other, providing flexibility in how clinicians use the filter to track during procedures [20]. Spider FX EPDs do not have this feature of moving independently of the delivery guidewire. The potential advantage of the independence of the filter and the guidewire allows the endovascular interventionalist to utilize atherectomy devices without the concern of filter movement and subsequent debris embolization. The independence of the filter and wire also allows for filter capture without the loss of wire position, offering the potential advantage of not losing wire position during filter capture with NAV6 and, therefore, allowing subsequent crossing of the lesion if needed. However, Emboshield NAV6 EPD pore sizes are larger than those of the SpiderFX, therefore making microembolization more apparent [21]. The basket size of the SpiderFX EPD is also larger, making it potentially more preferential during procedures where a greater number of debris is expected.

However, use of both the Spider FX and Emboshield NAV6 is common to prevent the complications of DE, regardless of FDA indication [22]. Due to the novelty of the Emboshield NAV6 EPD use in lower extremity lesions, there are limited published safety and efficacy data. Overall, the Emboshield NAV6 EPD exhibited similar effectiveness in comparison to the SpiderFX EPD, where no differences were observed in the individual types of MAEs between each group or the total number of MAEs in the dataset.

There were multiple limitations in the present study. Retrospective analysis of databases contains an inherent level of bias. This study was also based in a single center. Patients with critical limb ischemia were not included in this study, despite the potential benefit they may gain from distal protection. This decision was made because of the more pronounced clinical consequences observed when DE occurs in this population. It should also be noted that the atherectomy technique for each procedure could contribute to the risk of macroemboli, which can be minimized by avoiding fast cuts and advancing atherectomy devices quickly.

## 5. Conclusions

Emboshield NAV6 has demonstrated similar performance to the SpiderFX filter, based on exhibiting a high freedom from composite MAE rate and a low number of filter-related adverse events, meeting the initial performance goal based on historical data. These findings illustrate the safety and efficacy of both filter usages in the treatment of patients with symptomatic PAD.

## Figures and Tables

**Table 1:** Demographics and risk factors.

% (*n*/*N*)	Emboshield NAV6 (*N* = 161)	SpiderFX EPD (*N* = 346)	*p* value
Gender (female)	46.3% (75/161)	43.6% (151/346)	0.575
Age (years)^a^	69.8 ± 10.3 (161)	68.8 ± 10.8 (346)	0.844
BMI (kg/m^2^)^a^	27.3 ± 5.0 (161)	28.2 ± 5.7 (346)	0.416
Diabetes mellitus	59.3% (96/161)	61.8% (214/346)	0.577
Smoking	45.1% (73/161)	53.2% (184/346)	0.088
Hyperlipidemia	97.5% (158/161)	87.0% (301/346)	<0.05
Hypertension	98.1% (159/161)	94.8% (328/346)	0.077
Coronary artery disease	83.3% (135/161)	83.8% (290/346)	0.891
Chronic kidney disease	3.1% (5/161)	1.4% (5/346)	0.215

^a^Values in mean ± SD. ^b^By normal approximation for continuous variables and the Newcombe score method for binary variables. BMI, body mass index; HDL, high-density lipoprotein; LDL, low-density lipoprotein; SD, standard deviation.

**Table 2:** Baseline lesion characteristics.

% (*n*/*N*)	Emboshield NAV6 (*N* = 161)	SpiderFX EPD (*N* = 346)	*p* value
Lesion length (mm)^a^	110.4 ± 79.3 (161)	116.8 ± 76.3 (346)	0.549
RVD (mm)^a^	5.5 ± 0.6 (161)	5.6 ± 0.6 (346)	0.848
In-stent restenosis	6.8% (11/161)	8.1% (28/346)	0.607
Calcium	43.2% (70/161)	37.9% (131/346)	0.251
Chronic total occlusion	28.4% (46/161)	27.5% (95/346)	0.826
Run-off vessel grade			
0	14.8% (24/161)	24.3% (84/346)	
1	38.9% (63/161)	32.7% (113/346)	
2	27.8% (46/161)	25.7% (89/346)	
3	18.5% (30/161)	17.3% (60/346)	
Pooled	1.5 ± 1.0 (161)	1.4 ± 1.0 (346)	0.180

^a^Values in mean ± SD. ^b^By normal approximation for continuous variables and the Newcombe score method for binary variables. RVD, reference vessel diameter; SD, standard deviation.

**Table 3:** Procedural results.

% (*n*/*N*)	Emboshield NAV6 (*N* = 161)	SpiderFX EPD (*N* = 346)	*p* value
Directional (SilverHawk)	74.5% (120/161)	69.9% (242/346)	0.338
Rotational (Jetstream)	13.0% (21/161)	14.7% (51/346)	0.593
Laser (Turbo Elite)	13.0% (20/161)	15.3% (53/346)	0.483
Recoil	2.5% (4/161)	0.0% (0/346)	<0.05
Access complication	0.0% (0/161)	0.6% (2/346)	0.332
Stenosis (pre)^a^	90.3 ± 8.4 (161)	89.0 ± 11.0 (346)	<0.05
Stenosis (post)^a^	1.4 ± 5.5 (161)	1.6 ± 9.3 (346)	0.449
Thrombus present	1.2% (2/161)	0.9% (3/346)	0.696
Filter overflow	10.5% (17/161)	8.7% (30/346)	0.509
Perforation	0.6% (1/161)	0.0% (0/346)	0.144
Presence of microembolization	59.9% (97/161)	64.2% (222/346)	0.352

^a^Values in Mean ± SD. ^b^By normal approximation for continuous variables and the Newcombe score method for binary variables. SD, standard deviation.

**Table 4:** Major adverse events.

% (*n*/*N*)	Emboshield NAV6 (*N* = 161)	SpiderFX EPD (*N* = 346)	*p* value
Freedom from MAE	92.0% (149/161)	91.6% (317/346)	0.721
Death	0.6% (1/161)	0.3% (1/346)	0.582
MI (modified ARC)	1.2% (2/161)	2.9% (10/346)	0.252
TVR	0.0% (0/161)	0.6% (2/346)	0.332
Thrombosis	0.6% (1/161)	1.2% (4/346)	0.556
Dissection (grade C or greater)	4.9% (8/161)	2.9% (10/346)	0.245
Distal embolization	0.6% (1/161)	0.9% (3/346)	0.767
Perforation at the level of the filter	0.0% (0/161)	0.0% (0/346)	N/A
Unplanned amputation	0.0% (0/161)	0.0% (0/346)	N/A

^a^By the Newcombe score method. ARC, academic research consortium; MAE, major adverse event; MI, myocardial infarction; TVR, target vessel revascularization.

**Table 5:** Subset analysis of event rates by calcified lesions in the Emboshield NAV6 group.

% (*n*/*N*)	Calcified NAV6 (*N* = 70)	Noncalcified NAV6 (*N* = 90)	*p*	Calcified SpiderFX (*N* = 131)	Noncalcified SpiderFX (*N* = 216)	*p*
Freedom from MAE	88.6% (62/70)	94.6% (87/91)	0.167	95.4% (125/131)	91.7% (198/216)	0.264
Death	0.0% (0/70)	1.1% (1/91)	0.379	0.0% (0/131)	0.5% (1/216)	0.434
MI (modified ARC)	0.0% (0/70)	2.2% (2/91)	0.379	1.5% (2/131)	3.7% (8/216)	0.237
TVR	0.0% (0/70)	0.0% (0/91)	N/A	0.0% (0/131)	0.9% (2/216)	0.268
Thrombosis	0.0% (0/70)	1.1% (1/91)	0.379	1.5% (2/131)	0.9% (2/216)	0.615
Dissection (grade C or greater)	11.4% (8/70)	0.0% (0/91)	0.001	0.7% (1/131)	1.4% (3/216)	0.033
Distal embolization	0.0% (0/70)	1.1% (1/91)	0.379	0.7% (1/131)	0.9% (2/216)	0.871
Perforation at the level of the filter	0.0% (0/70)	0.0% (0/91)	N/A	0.0% (0/131)	0.0% (0/216)	N/A
Unplanned amputation	0.0% (0/70)	0.0% (0/91)	N/A	0.0% (0/131)	0.0% (0/216)	N/A

^a^By the Newcombe score method. ARC, academic research consortium; MAE, major adverse event; MI, myocardial infarction; TVR, target vessel revascularization.

**Table 6:** Subset analysis of event rates by chronic total occlusions in the Emboshield NAV6 group.

% (*n*/*N*)	CTO NAV6 (*N* = 46)	Non-CTO NAV6 (*N* = 115)	*p* value	CTO SpiderFX (*N* = 95)	Non-CTO SpiderFX (*N* = 251)	*p* value
Freedom from MAE	84.8% (39/46)	94.8% (109/115)	0.074	88.4% (84/95)	92.4% (232/251)	0.334
Death	0.0% (0/46)	0.9% (1/115)	0.526	0.0% (0/95)	0.4% (1/251)	0.538
MI (modified ARC)	2.2% (1/46)	0.9% (1/115)	0.113	5.3% (5/95)	2.0% (5/251)	0.105
TVR	0.0% (0/46)	0.0% (0/115)		0.0% (0/95)	0.8% (2/251)	0.383
Thrombosis	0.0% (0/46)	0.9% (1/115)	0.526	0.0% (0/95)	1.6% (4/251)	0.216
Dissection (grade C or greater)	10.9% (5/46)	2.6% (3/115)	0.030	5.3% (5/95)	2.0% (5/251)	0.105
Distal embolization	2.2% (1/46)	0.0% (0/115)	0.113	1.1% (1/95)	0.8% (2/251)	0.819
Perforation at the level of the filter	0.0% (0/46)	0.0% (0/115)	N/A	0.0% (0/95)	0.0% (0/251)	N/A
Unplanned amputation	0.0% (0/46)	0.0% (0/115)	N/A	0.0% (0/95)	0.0% (0/251)	N/A

^a^By the Newcombe score method. ARC, Academic Research Consortium; CTO, chronic total occlusion; MAE, major adverse event; MI, myocardial infarction; TVR, target vessel revascularization.

## Data Availability

Data are available upon request.
